# Extreme subsidence in a populated city (Mashhad) detected by PSInSAR considering groundwater withdrawal and geotechnical properties

**DOI:** 10.1038/s41598-020-67989-1

**Published:** 2020-07-09

**Authors:** Mohammad Khorrami, Saeed Abrishami, Yasser Maghsoudi, Babak Alizadeh, Daniele Perissin

**Affiliations:** 1 Department of Civil Engineering, Faculty of Engineering, Ferdowsi University of Mashhad, Mashhad, Iran; 2 Department of Civil and Coastal Engineering, University of Florida, Gainesville, FL 32611 USA; 3 Department of Photogrammetry and Remote Sensing, Faculty of Geodesy and Geomatics Engineering, K. N. Toosi University of Technology, Tehran, Iran; 4 Institute of Geophysics and Tectonics, University of Leeds, Leeds, UK; 5 Department of Civil Engineering, University of Texas At Arlington, Arlington, TX 76019 USA; 6 RASER Limited, Hong Kong, P. R. China

**Keywords:** Hydrology, Natural hazards

## Abstract

Ground deformation can cause serious environmental issues such as infrastructure damage, ground compaction, and reducing the ground capacity to store water. Mashhad, as one of the largest and most populated cities in the Middle East, has been suffering from extreme subsidence. In the last decade, some researchers have been interested in measuring land subsidence rates in the Mashhad valley by InSAR techniques. However, most of those studies were based on inaccurate measurements introducing uncertainties in the resulting subsidence rates. These researches used a small number of EnviSat data with long perpendicular and inhomogeneous temporal baseline. This paper seeks to determine the subsidence rate in urban areas of Mashhad in recent years, the threat that was neglected by the city managers and decision-makers. For this purpose, the Persistent Scatterer InSAR technique was applied in the study area using two time-series of descending and ascending Sentinel-1A acquisitions between 2014 and 2017. The results demonstrated the maximum line-of-sight deformation rate of 14.6 cm/year and maximum vertical deformation (subsidence) rate about 19.1 cm/year which could have irreversible consequences. The results were assessed and validated using piezometric data, GPS stations, and geotechnical properties. This assessment confirms that the main reason for subsidence in the interested area is groundwater over-extraction. Also, investigation of geotechnical properties shows that thick fine-grained layers in the northwest of the city could strongly affect the results. At the end of this paper, a new simplified method was proposed to estimate specific storage in special cases to predict the subsidence rate.

## Introduction

Ground deformation has been reported as one of the severe geological hazards around the world^1^. It is mainly due to the fact that anthropogenic activities such as fluid extraction or injection, underground excavations, and expanding construction have been increased in many regions as a forthcoming problem^2–5^.

Multiple studies have reported that several areas are suffering from land subsidence as a result of fluid over-extraction. These areas include but are not limited to cities in China, Indonesia^6^, Italy^7,8^, Mexico^9,10^, the United States^11–15^, Greece^16^, Belgium^17^, Brazil^18^, Spain^19^, and Taiwan^20^. Likewise, there are many regions in Iran suffering from groundwater overexploitation and corresponding land deformations. For instance, groundwater drawdown led to extreme ground displacement in large cities in Iran including Tehran, Rafsanjan, Neyshabour, and Mashhad^21–31^.

The present study focuses on the large subsidence velocities of the urban areas in Mashhad, Iran, which can significantly enhance the results of previous studies. Lacking sufficient natural recharge in the past decades as well as groundwater over-extraction due to urbanization, population growth, tourism industry, and inappropriate groundwater management has resulted in severe groundwater level reduction and local subsidence in many areas of the Mashhad Valley, where Mashhad city is located in. A preliminary analysis^21^ in Mashhad Valley conducted approximately 8 km northwest of Mashhad, reported the results of leveling, GPS monitoring, and Interferometric Synthetic Aperture Radar (InSAR) analysis and showed the maximum line-of-sight (LOS) deformation rate of 28–30 cm/year between 2003 and 2005. Another investigation^32^ analyzed the same study area using the Small Baseline Subset (SBAS) algorithm and reported the maximum LOS deformation rate of 23 cm/year between 2003 and 2005. These studies have mentioned groundwater as the main reason for the high rate of subsidence. Another research conducted in this region^22^ demonstrated an annual LOS subsidence rate of 25 cm/year, from 2004 to 2007, using the InSAR method. Furthermore, a study^33^ reported a 65 m water level drawdown during four decades in some parts of the valley. This groundwater drawdown resulted in subsidence emerging in some parts of the area, particularly agricultural regions.

There are several well-known and highly-used land surface subsidence measurement methods. Among these methods, SAR interferometry is proven as a powerful technique in mapping deformations of both agricultural and urban areas. This method is more accurate applicable than in-situ and geodetic techniques, such as leveling and GPS, as it can effectively provide an opportunity to achieve extensive ground deformation^34,35^. By applying InSAR processing techniques such as Persistent Scatterers Interferometric-SAR (also known as PSInSAR, PSI, and PS)^36^ to a dataset of radar images over a specific region, it is possible to obtain ground surface movements with the high precision i.e. millimeters.

This work presents the results of the PSI time series analysis obtained by the exploitation of Sentinel-1 SAR data archives between October 2014, and February 2017, to investigate land subsidence in Mashhad. Sentinel-1 is a two-satellite constellation with the prime objectives of Land and Ocean monitoring to provide C-Band SAR data continuity following the retirement of ERS-2 and the end of the EnviSat mission. The data are relatively accessible in most areas, the coverage is temporally frequent and spatially large with the moderate spatial ground resolution^37^.

In the case of SAR data, previous studies on Mashhad used EnviSat data for InSAR processing. From literature^38,39^ it is indicated that such data would have low coherence for mixed land use of vegetation and buildings. It is reported^40^ that the coherence for C-band SAR can be well conserved if both the perpendicular and temporal baseline are small. The coherence values in the interferograms decrease with increasing the perpendicular and temporal baseline because of the time span between the images acquisitions^41^. Long perpendicular baseline causes serious geometric decorrelation and the Digital Elevation Model (DEM) errors resulting in a negative impact on the accuracy of deformation estimation^42–45^. Additionally, it is demonstrated that homogeneous temporal baselines give more accurate results^46^. A recent study^47^ observed that the interferograms with large temporal baseline (i.e., > 22 days), as seen in InSAR analysis on EnviSat data, were decoherent. Therefore, the phase unwrapping can not be performed correctly, and useful information is hardly extracted from them. Such crucial problems and decorrelations can be reduced with finer imaging resolution and shorter temporal baseline. Sentinel-1 SAR data has the advantages of shorter revisit time and finer imaging resolution resulting in the improvement of the derived coherence value. It should be noted that EnviSat data may be used to clearly identify the location of subsidence and may be useful for some initial analysis even in urban areas; however, the high phase gradient over a small spatial extent made the phase fringes undistinguishable. Thus, due to the large baseline distances in some pairs, the interferograms are relatively noisier than the others and finally, high phase noise makes the phase unwrapping very difficult.

In the past decade, some researchers have been interested in measuring land subsidence rates in the Mashhad valley by using InSAR techniques. However, most of those studies were based on inaccurate measurements introducing uncertainties in the resulting subsidence rates. These researches used a small number of EnviSat data with long perpendicular and inhomogeneous temporal baseline. From this point of view, in the present study compared to the previous studies on Mashhad, we: (a) used Sentinel-1 SAR data which provided finer imaging resolution, and smaller and homogeneous temporal separation, (b) used both ascending and descending mode, (c) applied PSI technique and focused mainly on urban areas to obtain a high density of Persistent Scatterers to ensure that the obtained land subsidence results would be more accurate, (d) computed line-of-sight and vertical subsidence, and (e) calculated the subsidence rate in recent years to update the previous studies.

The intellectual merit of this paper is the focus on the combination of InSAR and piezometric data in specific storage estimation which can help the subsidence estimation and prediction. We used the specific elastic storage concept to help the subsidence prediction in the study area. In the previous works estimating the specific storage from land subsidence, continuous measurements of compaction from extensometers^48–50^, and InSAR^2,51,52^ in combination with time-series groundwater level measurements from neighboring piezometers have been used to calculate specific storage coefficients of aquifer systems. Also, drainage of groundwater in soil deposits can induce huge land subsidence. Therefore, the other significance of this research is to investigate deep geotechnical wells to detect thick compressible sediments in the areas suffering from groundwater extraction.

This paper is organized as follows. First, the study area is introduced including information such as its location and water demand. Second, a brief description of the basic concepts of PSI and the dataset is given. Third, PSI is applied to produce a subsidence map using SARPROZ software. Fourth, piezometric and GPS data is provided to assess the subsidence map. Finally, soil properties are presented for deep wells to estimate specific storage in our new proposed method.

### Study area

Mashhad, covering an area of 311 km^2^, is the second most populated city in Iran, dominated by semi-arid climate^32^ with the average annual precipitation of 250 mm^53^. Based on recent research on the municipal water demand in Mashhad^54^, the annual water demand of the city is almost 220 million cubic meters and its daily water consumption fluctuates between 270 and 830 thousand cubic meters annually. The considerable fluctuations in the water demand may be due to its 20 million annual visitors which causes huge water consumption^54^.

## Methodology

### Line-of-sight deformation time-series generation by PSI

In this paper, we applied the PSI technique^36^, an extension of the conventional InSAR, to monitor ground deformation over urban areas of Mashhad. PSI technique is a powerful radar-based remote sensing method in the evaluation of land subsidence over a period, particularly in urban areas^55,56^. By implementing this technique, it can be possible to monitor the temporal and spatial evolution of subsidence at millimeter precision and produce mean deformation velocity maps^57^. The technique selects a number of points (or targets) known as Persistent Scatterers (PSs). The PSs, which are on the ground surface, should have stable scattering properties, decent temporal coherence, and stable behavior within a period^58^. To determine the subsidence rate, several SAR images of the same area are required. Then, one master acquisition is chosen from the dataset, considering baselines in time and space, called temporal baseline and spatial baseline. Preferably, the master image is selected in the middle of the spatial and temporal baseline spaces^59^. Afterward, the co-registered Single Look Complex (SLC) images are created using this single master configuration. Finally, the earth curvature and the topography influence is removed from the phase to acquire a series of phases for each pixel to obtain deformations.

Several factors influenced the phases, such as linear phase ramp, external DEM inaccuracy, atmospheric, the scatterer movement, and decorrelation noise as in the following equation^60^.

1 $${\varphi }^{k}= \left(\frac{4\pi }{\uplambda }\times \frac{{B}_{\perp }^{k}}{R\mathrm{sin}\theta }\times h\right)+ \left(\frac{4\pi }{\uplambda }{\times T}^{k}\times v\right)+{\varphi }_{atm}^{k}+{\varphi }_{orb}^{k}+{\varphi }_{noise}^{k}$$

where the first term is related to the DEM error (*h*) because of the external DEM inaccuracy, the second one denotes the linear deformation velocity (*v*) stemming from the displacement of the target scatterers (scatterer movement) during the acquisition period. ${B}_{\perp }^{k}$ is the normal (perpendicular) baseline value of the k-th interferogram. *R* denotes slant range, *T* refers to time, and $\uplambda$ is the wavelength. ${\varphi }_{atm}^{k}$, ${\varphi }_{orb}^{k}$ and ${\varphi }_{noise}^{k}$ denote the atmospheric phase delay, the residual orbital error phase, and the decorrelation noise (temporal and geometrical decorrelations), respectively.

There is an important fact that PS values are relative to a stable reference point, chosen within the PS dataset^61^. In this study, it is assumed that the ground deformation rate is linear as the standard approach. It should be noted that high spatial densities of PS points correspond to urban environments, even though low spatial densities are found in agricultural areas because of temporal decorrelation^62^ in these locations.

Subsidence monitoring by PSI in our study was conducted by SARPROZ^63^, and the applied processing steps are as follows^64^:

In Step 1, the PS Candidates (PSCs) are selected: each pixel could be a PSC if it satisfies the following condition for the amplitude stability index.

2 $${D}_{stab}=1-\frac{{\sigma }_{a}}{\stackrel{-}{a}}\ge 0.7$$

where $\stackrel{-}{a}$ is the mean of amplitude values calculated at each pixel from the time series and ${\sigma }_{a}$ is the standard deviation related to variation of amplitude in time.

In Step 2, unknown parameters are estimated: based on relative phase information between nearby PS, the unknown parameters of DEM error and the velocity are estimated. For this purpose, the spatial graph of connections between points is considered and the initial parameters are estimated along with the connections. Then, the absolute values are achieved in the way of a numerical integration that is calculated considering a reference point as a starting point for the integration. Choosing the reference point plays an important role in the accuracy of results since an undesirable reference selection might result in biased parameters for all points.

In Step 3, subsidence rates are estimated: after the APS removal, based on a spatial coherence condition, a wider set of points are selected. Spatial smoothing designed considering a correlation distance of about 500 m and high pass filter in time domain based on decorrelation at the time sampling frequency. Then, a second approximation of the parameters is implemented on the new dataset. Finally, considering a threshold for temporal coherence, all PSs above the threshold are selected. To clarify, the threshold used for the final selection and analysis of PS points was based on the amplitude stability index and then the temporal coherence value of 0.8 is used as the last filter to show the most reliable points in the subsidence map. The DEM error, the linear displacement rate along the line-of-sight, and the deformation time series are approximately calculated for the selected PS points using a linear trend assumption. It should be mentioned that in this study DEM SRTM 30 m has been subtracted from the data. The process of estimating the rate and the height acts as the unwrapping process in the time domain. For this estimation, some assumptions and initial values (search space) are needed. So, the range of the parameters (initial values) inputted for estimating velocity and height were − 150 to 150 mm/year and − 50 to 50 m, respectively. The periodogram technique is then estimating the unknown parameters within the given range.

In order to compare the PSI and GPS data, we used the following equation to obtain GPS measurements in LOS direction^59^.

3 $${GPS}_{LOS}={GPS}_{up}\times \mathrm{cos}{\theta }_{inc}-{GPS}_{north}\times \mathrm{cos}{(\theta }_{azi}-\frac{3\pi }{2})\times \mathrm{sin}{\theta }_{inc}-{GPS}_{east}\times \mathrm{sin}{(\theta }_{azi}-\frac{3\pi }{2})\times \mathrm{sin}{\theta }_{inc}$$

where $GPS_{LOS}$ is the converted value of GPS data in LOS direction. ${GPS}_{up}$, ${GPS}_{north}$, and ${GPS}_{east}$ are the values of GPS observation vector in the up, north, and east directions. ${\theta }_{inc}$ represents incidence angle. The images were taken from different incidence angles and the average incident angles are about 43.96° and 39.22° in this study for ascending and descending tracks, respectively. Also, ${\theta }_{azi}$ represents the heading angle of the satellite from the north (azimuth angle) and is about − 170.43° and − 10.12° in this study for ascending and descending tracks, respectively.

### Decomposition of LOS displacement into vertical displacement rate

The subsidence signal in the Mashhad plain is covered by SLC data from two different ascending and descending orbits and therefore only two different viewing geometries are available. Consequently, only two of the three components of the actual displacement vector can be retrieved. The mean PS-InSAR LOS velocity is decomposed into east–west and vertical components. It is assumed that the north–south deformation was negligible because the near-polar orbits of the SAR satellites produce a low sensitivity to the north–south component of deformation^65^. Before decomposition, the InSAR mean velocity fields of both tracks are transformed into the same reference frame using a reference area considered as a stable area. Finally, the LOS velocity was decomposed into the horizontal component along the east–west direction (*v*_*hor*_) and the vertical component (*v*_*ver*_), considering the local incidence angle of the satellite view by solving the following equation^24^.

4 $$\left(\genfrac{}{}{0pt}{}{{v}_{asc}}{{v}_{desc}}\right)=\left(\genfrac{}{}{0pt}{}{\mathrm{cos}{\theta }_{asc} -\mathrm{ cos}{\alpha }_{asc}\mathrm{sin}{\theta }_{asc}}{\mathrm{cos}{\theta }_{desc}-\mathrm{ cos}{\alpha }_{desc}\mathrm{sin}{\theta }_{desc}}\right)\left(\genfrac{}{}{0pt}{}{{v}_{ver}}{{v}_{hor}}\right)$$

where ${\theta }_{asc}$ and ${\theta }_{desc}$ represent the local incidence angles and ${\alpha }_{asc}$ and ${\alpha }_{desc}$ are the satellite heading angles in the ascending and descending modes, respectively.

### SAR data description

To analyze the subsidence by PSI, it is essential to provide a sufficient number of SAR data. From literatures^60,66^, the PS analysis requires at least 20 to 25 SAR images. The availability of ascending and descending data is a significant factor in retrieving more accurate deformation rates. So, the dataset under study composed of 69 Sentinel-1 SLC images including 34 descending and 35 ascending images acquired between October 2014 and February 2017. Table 1 shows a brief overview of the SLC data used in the PSI processing procedure. To start subsidence monitoring, we introduced the study area for each track of data (descending and ascending). Table 2 provides detailed information of the SLC data of these areas including the incidence angle and the satellite heading angle in the ascending and descending modes.

**Table 1:** SLC data used in the PSI processing procedure.

Sensor	Track	Mode	Polarization	# Scenes	Master image acquisition date
Sentinel-1A	86	Ascending	VV	35	03-02-2016
Sentinel-1A	93	Descending	VV	34	15-06-2016

**Table 2:** Detailed information about SLC data of the areas of study (shown in Fig. 1).

Area	Mode	Sub-swath	Mean incidence angle	Mean heading angle
1	Descending	2	39.1985^o^	− 10.1123^o^
2	Ascending	2	43.9594^o^	− 170.4323^o^
3	Descending	1	39.2224^o^	− 10.1152^o^

**Figure 1:**
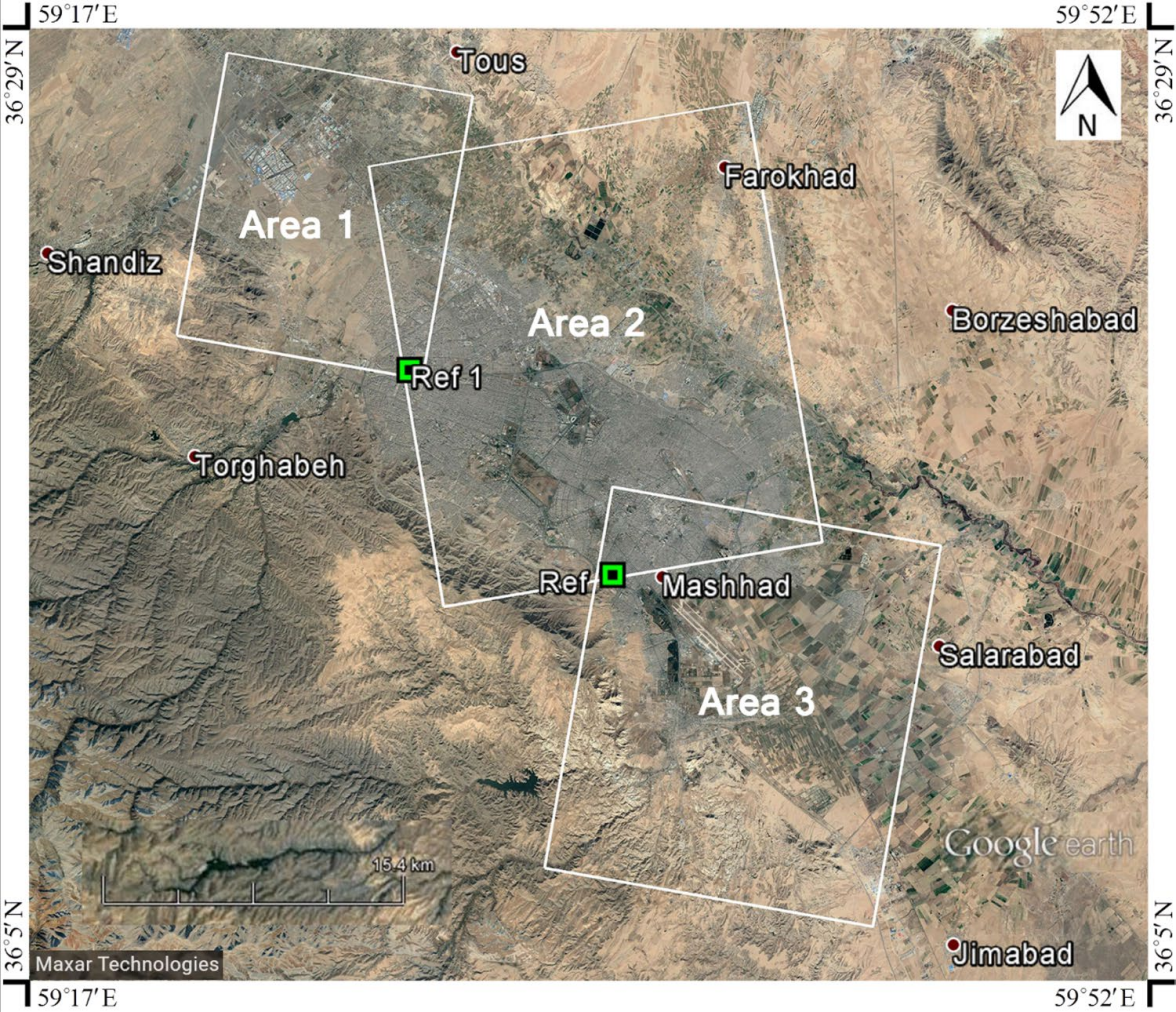
Study area: Area 1 (descending), Area 2 (ascending), and Area 3 (descending). The reference points are shown by the green squares. Ref is the location of the main reference point.

As seen in Fig. 1, we had one ascending area and two descending areas and so, it was not possible to do one analysis. Thus, three separate areas were chosen to cover the areas of interest and the analysis was done on three separate sub-swaths of sentinel data. Accordingly, it was required to introduce a reference point for each analysis. In order to have the same reference for all three areas, one main reference point was defined in Area number 2 (Ref point), and the reference points for Area 1 and Area 2 were selected based on the main reference point. It means the first analysis was done in the central area (Area 2) which was the largest area covering some parts of Area 1 and Area 3. Besides, the reference points required for the analysis of Area 1 and Area 3 were selected in areas covered by Area 2 having the least amount of displacement rate compared to the main reference. To obtain more accurate outputs, we used the same reference point for Area 2 and 3 (Ref) and one reference for Area 1 (Ref 1). The reference point was controlled in the area of interest to evaluate if it really corresponds to the geologically motionless point and also based on spatial coherence and temporal coherence. So, PS datasets were processed using the main reference point not affected by rapid deformation. Based on the preliminary data analysis and geological prospection for the reference point (stable or zero displacement), the location of the reference point is chosen.

**Figure 2:**
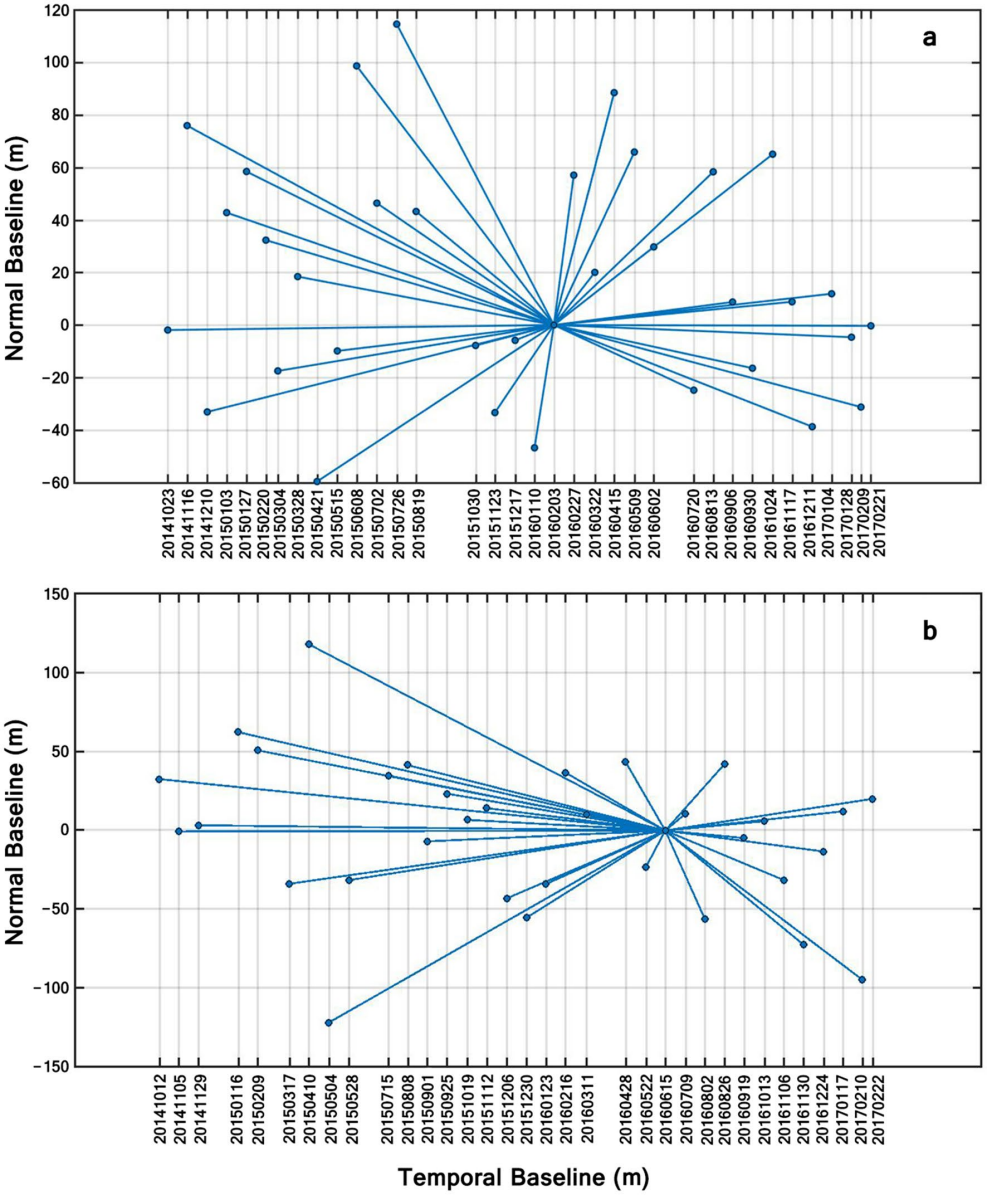
The spatiotemporal baseline configuration of interferometric pairs: (**a**) Ascending images, and (**b**) Descending images.

The spatiotemporal baseline configuration of interferometric pairs is shown in Fig. 2. To form interferograms, all images were connected with the master image, which is chosen at the barycenter of the temporal baseline (x-axis) and normal baseline (y-axis) distributions. The dots represent the images and the lines represent the interferograms.

## Results and discussion

### Subsidence map of the study area

**Figure 3:**
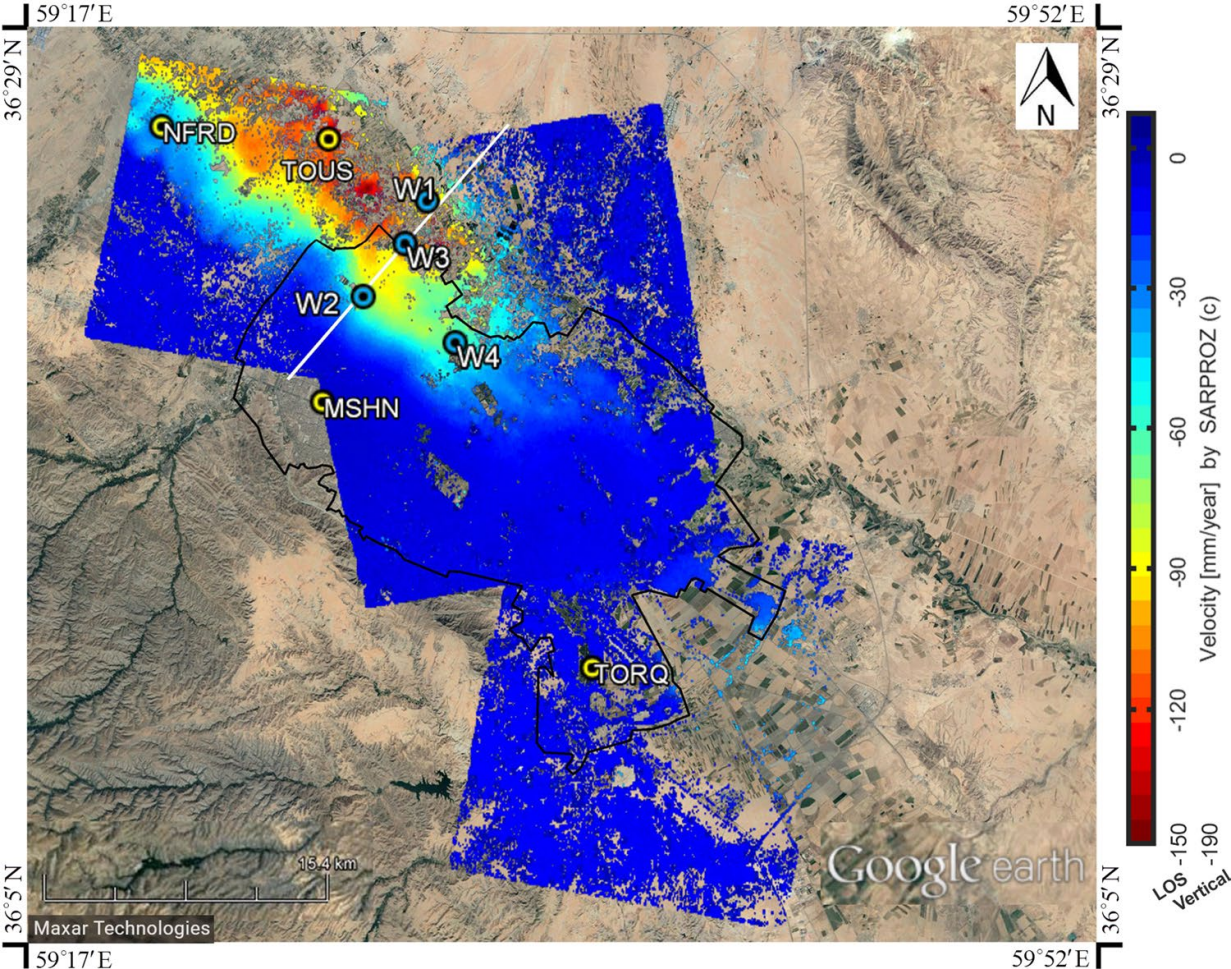
Mean velocity map of land subsidence in the Mashhad region covering a period between Oct. 2014 and Feb. 2017 and the location of GPS stations (MSHN, TORQ, NFRD, and TOUS) and piezometric wells (W1 to W4).

Implementing PSI in the study area resulted in a map of the line-of-sight displacement rate. Figure 3 shows the average velocity map of LOS deformation in Mashhad calculated from Oct. 2014 to Feb. 2017. Based on the spatial coherence of the PSs calculated in the area of interest, most of the area is covered by the coherence of 0.80 or higher. Thus, the coherence of the map proves the reliability of the subsidence monitoring process by PSI. In addition, a separate analysis of ascending and descending data shows the same deformation pattern which validates the implementing technique and the outputs (cross-validation). Vegetation areas include less coherent PS points; thus, there are some regions without sufficient outputs in the deformation map.

From the extracted map it can be concluded that northwest of the city is suffering from a high range of subsidence with a maximum LOS deformation rate of 14.6 cm/year and maximum vertical deformation (subsidence) of 19.1 cm/year close to TOUS GPS station. The red spots in Fig. 3 indicates the maximum displacement rate area. There are studies^67,68^ showing an increase in groundwater level in some parts of Mashhad, particularly since 2008 due to transferred water (from Doosti dam) to the town and expansion of sewage network in the region. So, as a hypothesis, it may be a reason to slightly reduce the subsidence rate during the last decade.

In order to evaluate the outputs of PSI analysis, the GPS observations and four well points (for groundwater monitoring and soil properties investigation) are considered in the present study and their locations are shown in Fig. 3. The characteristics and information of the wells are fully explained in the following sections.

### GPS monitoring

GPS data has a high temporal resolution because of continuous measurements while the PSI method provides high spatial resolution and lower temporal resolution compared to data from GPS stations. Thus, the integration of GPS and PSI measurements can be used to interpret the land displacements. To assess the PSI results in the present work, all GPS stations neighboring Mashhad are investigated. Table 3 shows the start date, the location, and the current situation (active/inactive) of the GPS stations. It should be noted that there is not a sufficient number of GPS stations in the area to monitor the land displacements.

**Table 3:** GPS stations neighboring the interested area.

GPS station	Start date	Location		Current situation
		Long	Lat	
MSHN	Dec. 21, 2004	59.4797	36.3344	Active
NFRD	Sep. 19, 2007	59.4013	36.4501	Active
TORQ	Apr. 24, 2005	59.6272	36.2239	Active
TOUS	Dec. 23, 2004	59.4889	36.4450	Inactive since 2012

**Figure 4:**
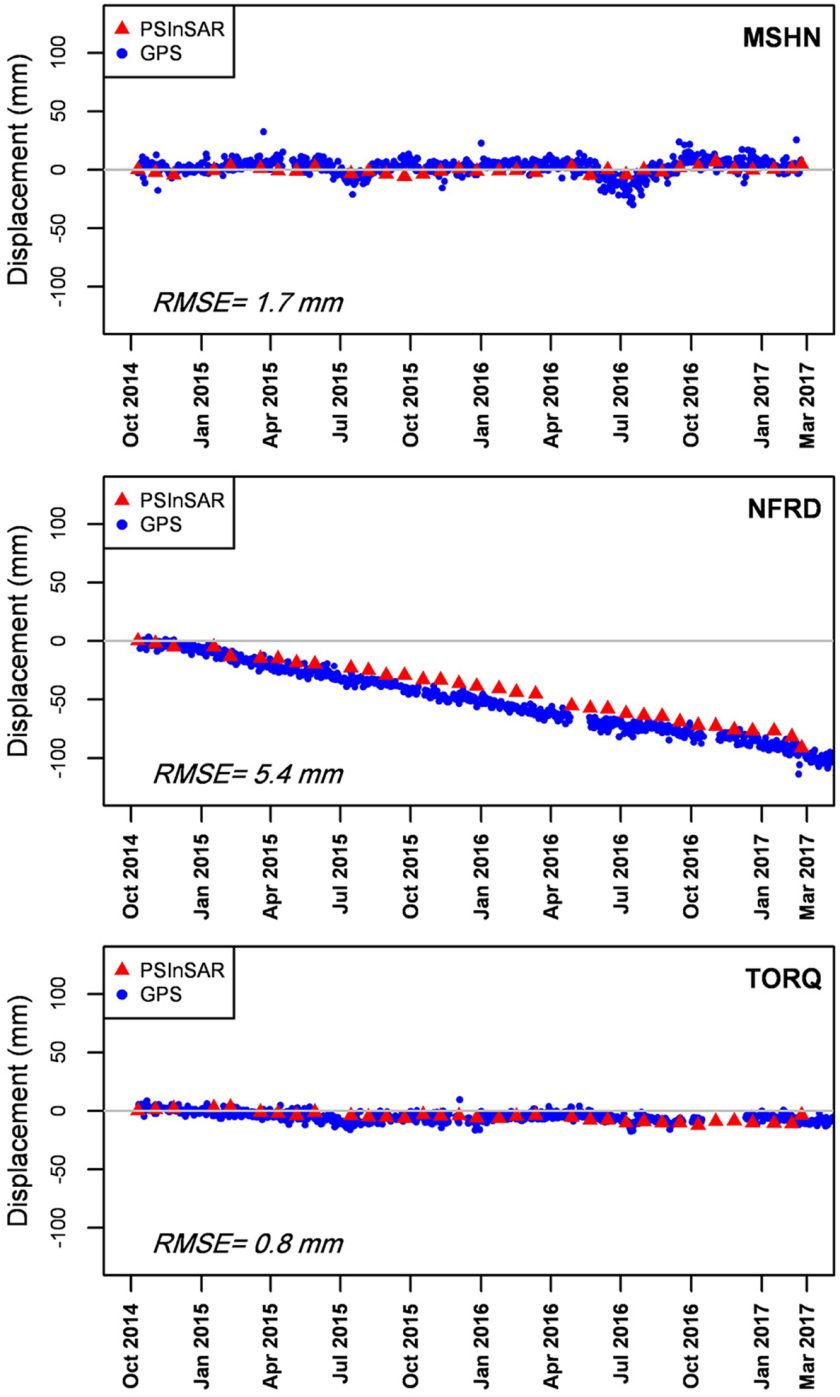
Comparison between PSI results and GPS observations projected into LOS displacement.

Figure 4 shows the comparison between the PSI-derived displacement time series and the corresponding observations of GPS stations in LOS direction. For this purpose, the root mean square error (RMSE) is calculated between PSI output and GPS observation and demonstrated their relatively decent agreement.

**Figure 5:**
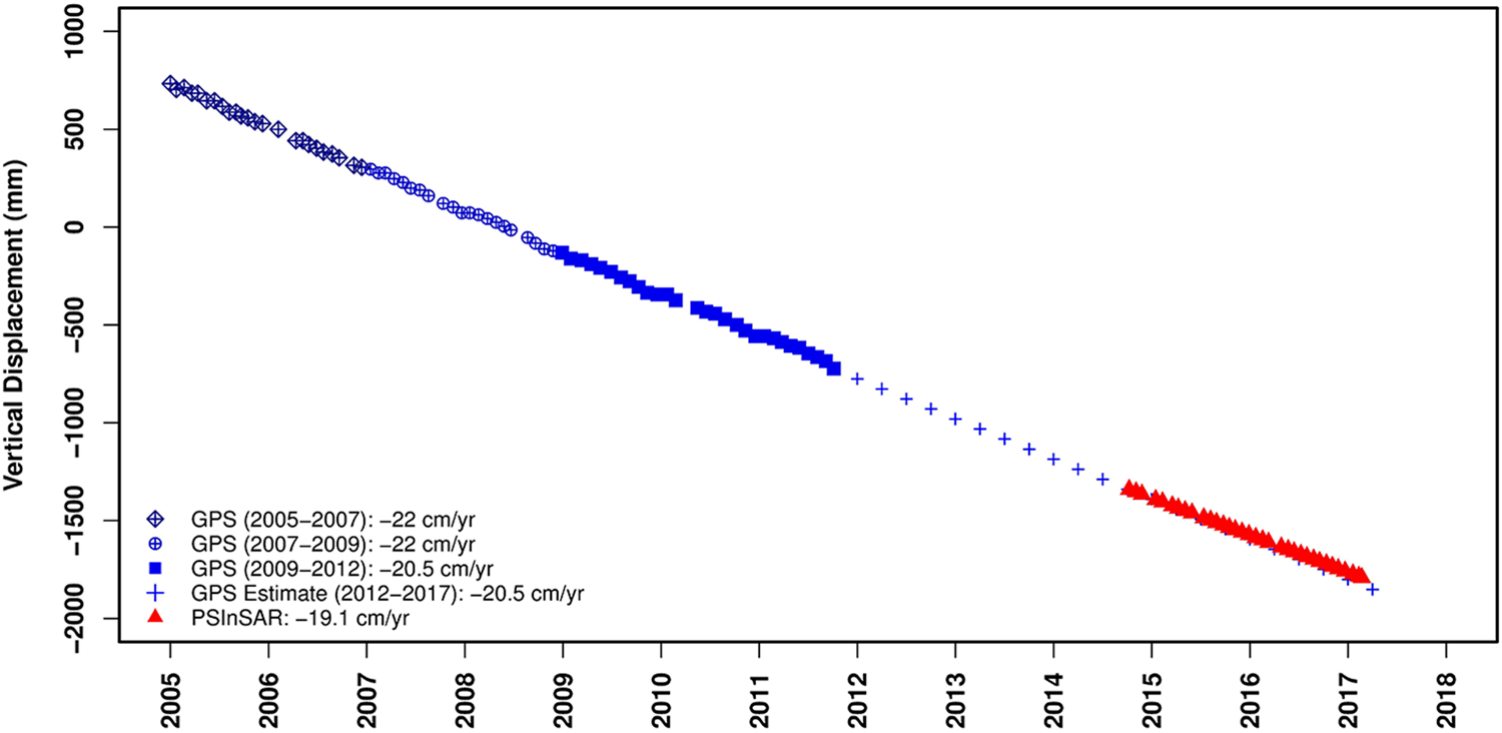
Comparison between vertical displacement obtained by PSI technique and TOUS GPS Station.

As shown in Fig. 5, the vertical deformation rate observed by TOUS station was approximately constant between 2005 and 2009 (22 cm/year), and then, this value slightly reduced to 20.5 cm/year from 2009 to 2012. The TOUS station is located close to the area with the maximum deformation rate. This station was inactive since 2012; therefore, as its trend was approximately linear in a decade, we assumed a linear trend for its observation from 2012 to 2017 (GPS Estimate) which is in a good agreement with the result of our study (19.1 cm/year).

Lacking a sufficient number of GPS stations is a substantial weakness of GPS stations in monitoring ground displacements compared to the SAR Interferometry technique. Furthermore, the fluctuations in GPS outputs referred to seasonal effects and the inherent errors of instrument^69^. These are the main drawbacks or weaknesses of GPS measurements compared to the InSAR techniques. It is worth mentioning that despite the various advantages of InSAR technology compared to the GPS data such as its high spatial coverage, the GPS stations are still essential and cannot be totally replaced by this technology. First of all the technique can only provide the deformation in two directions and assumes a zero north–south deformation which is not valid in many cases. Secondly, the GPS data are an inseparable part of many applications that employ the InSAR derived velocities to estimate the strain rates. Last but not least, technology is still flourishing and every day we can see new researches to improve the technique. All these researches require GPS data for their validation.

### Groundwater level variations

**Figure 6:**
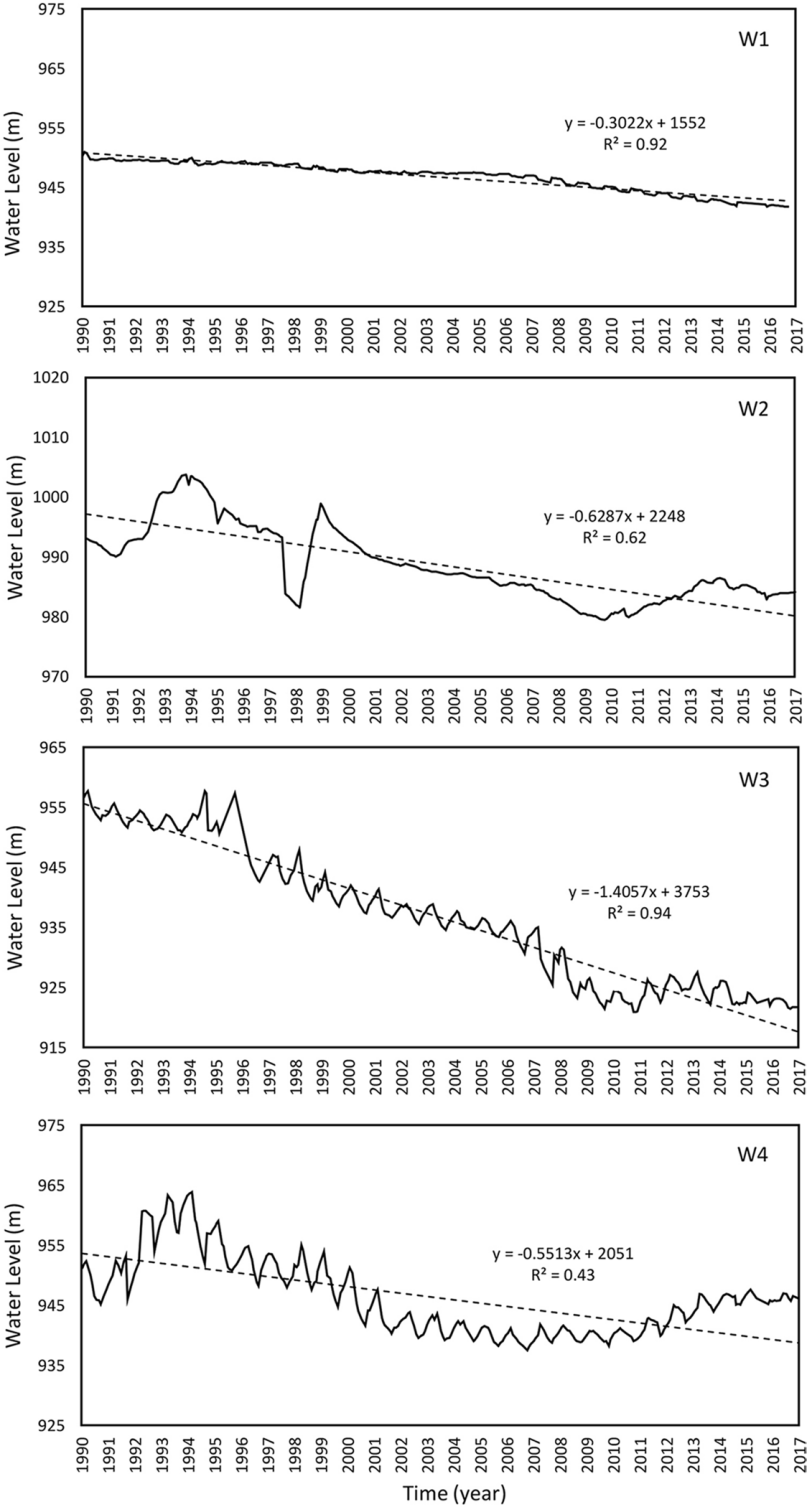
Piezometric level temporal evaluation from 1990 to 2017 at four selected well points (W1, W2, W3, and W4). Continuous line and dashed line indicate the groundwater level change and the trend line of piezometric level, respectively.

As a result of huge water demand, the groundwater level in the study area has been decreasing in the last decades. From the previous researches and reports on Mashhad, discussed in the literature, the main reason for the subsidence in this area is groundwater withdrawal. Therefore, we collected the water level variations of piezometric wells to assess the effect of groundwater level variations on the subsidence rate in the interested area (northwest of Mashhad). Figure 3 displays the location of the piezometric wells (W1, W2, W3, and W4). The groundwater level changes, from 1990 to 2017, in the four wells are presented in Fig. 6. Trend lines (dashed lines) are plotted to obtain the overall situation of water level variations in each well.

Investigating the water level variations, shown in Table 4, the maximum water level decrease occurred in W3 (around 35 m) and the maximum subsidence rate, obtained by the PSI analysis, is observed in the location of W3 (140 mm/year). Surprisingly, the minimum subsidence rate, per one-meter water level decrease, is also observed in W3. This demonstrates the extent that the geotechnical properties can affect the amount of ground compaction.

**Table 4:** Overview of the water level variations in the piezometric wells.

Well no	Water level in 1990 (m)	Water level in 2017 (m)	Total water level decrease (m)	Average water level decrease^a^ (m/year)	Subsidence rate by PSI (mm/year)	Subsidence rate per 1 m decrease in water level (mm/year)
W1	950.3	941.7	8.5	0.30	78.5	9.2
W2	993.1	984.2	8.9	0.63	61.3	6.9
W3	956.5	921.7	34.9	1.41	140.0	4.0
W4	951.1	946.1	5.0	0.55	55.7	11.2

^a^Graph gradient based on the trend-line for each well in Fig. 6.

Thus, there are a few important points to note:

- A comparison of W3 with W1 and W2 shows that the thickness of the fine-grained layer at the W3 site is about 90 m. However, it is around 100 m and 110 m in W2 and W1, respectively. Therefore, it is expected to see a lower “subsidence rate per meter decrease in water level” in W3.
- The information on soil layers is just available up to a depth about 220 m, while according to other studies the alluvial thickness in this area is more than 350 m and no further information is available. This information may include more fine-grained thicknesses in W1 and W2.
- As the water level drops in W3, the top layer becomes unsaturated, and therefore, the thickness of the saturated fine-grained soil decreases. Considering the groundwater level between 2014 and 2017, the thickness of the fine layer was about 70 m.
- The existing lithology is very general and just separates fine and coarse grains; however, geotechnical characteristics of fine grains are unknown.
- Soil compaction does not change linearly or constantly and depends on the applied stress and current soil condition which by changing the stress, these parameters will also change^70^. In addition, the amount of consolidation is a function of the permeability of the consolidating soil, compressibility (the coefficient of volume compressibility), layer thickness, and boundary conditions (e.g. length of the drainage path). The coefficient of volume compressibility varies depending on the amount of clay, soil plasticity, and soil porosity^71^. Unfortunately, such information is currently not available in the area of interest, and this study emphasizes the vital need to collect such features in addition to general soil properties.

Therefore, the present study highlights and extracts this ambiguity by extracting the Ss parameter as explained in the next sections.

### Geotechnical and geological considerations

Geotechnical properties of the soft sediments in the area of interest are influenced by the geological condition. Inadequate or false knowledge of the geological and geotechnical conditions of an area leads to major risks^72–74^ such as detecting the locations with a risk of subsidence as a result of groundwater level changes. So, in this section, we study the overall geological and geotechnical characteristics of the interested area.

Mashhad is located in the northern slopes of Binalood Mountains and Paleo-Tethys suture zone and is mostly laid on thick Quaternary deposits surrounded by Marls in northeastern margins, Ultramafic and Mafic and outcrops in the southern parts, and Phyllite and Slate in the western and southwestern. Furthermore, Kashafrood River passing through the northeast of the city has important effects on the depositional process of the fine-grained deposits and is the most significant geomorphic phenomenon of Mashhad watersheds^53,75^. Based on the previous reports^53,75–77^, sediment and alluvial fan deposits generally contain gravels and sands, extend over the south, southwest, and west parts of the city and alluvial plain deposits consist of clays and silts, cover center and eastern parts.

**Figure 7:**
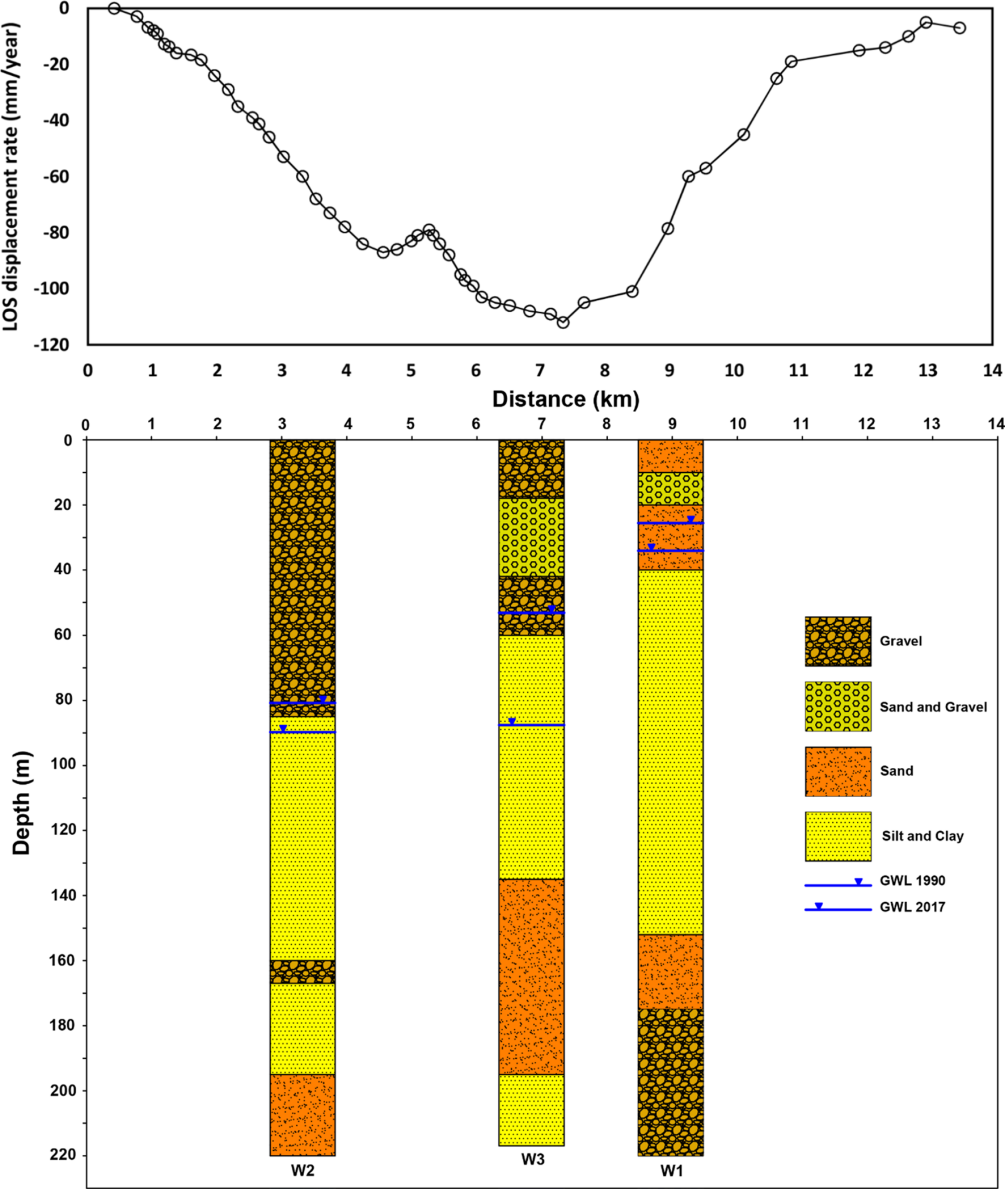
Line-of-sight displacement rate, obtained by PSI, along the A-A section (shown in Fig. 3, from west to east) and the lithological columns of deep wells in the study area.

Figure 7 shows ground displacement rate along the A-A section (shown in Fig. 3), from west to east, and the lithological columns of deep wells at the location of piezometric wells in the study area. Collecting deep geotechnical boreholes indicates thick fine-grained layers in the northwest of the city which have not been reported by past researchers. This finding confirms the high subsidence rates in the northwest of Mashhad due to a large amount of water extraction and corresponding consolidation phenomenon in fine-grained layers. Figure 7, also, demonstrates that the first 40 m, in-depth, of all boreholes, include coarse-grained materials. Accordingly, ordinary geotechnical investigations (common tests required for the construction of buildings) do not encounter thick fine-grained layers. This indicates the necessity of more detailed geotechnical investigations in the area of interest.

### Subsidence computation based on specific storage concept

While the InSAR method provides a highly accurate subsidence rate for a large study area, its application requires specific SAR images as well as the knowledge of utilizing them. Obtaining an initial estimation of subsidence rate using the geotechnical properties can be significantly helpful for the prediction of future soil behavior. In order to model subsidence caused by fluctuations in groundwater level, we used the concept of specific storage, i.e. the capacity of groundwater release, as a one-dimensional model. This approach is based on the assumption that deformations are caused only by vertical effective stress changes derived from groundwater level variations (piezometric data).

In geotechnical engineering, soil vertical deformation (land subsidence) is estimated by considering the following parameters: (a) deformable soil thickness, (b) effective stress variation, and (c) modulus relating the two previous parameters. The changes in stress state are due to the variations in the groundwater level. Piezometric levels were measured frequently and they are used to determine the groundwater table depth. It is important to note that pore water pressure changes are assumed to be the same as changes in the groundwater table. In this paper, we assume that the settlements are only caused by changes in pore water pressure. So, the variation of effective stress can be written as^70^:

5 $${\Delta \sigma }_{z}^{^{\prime}}=-{\gamma }_{w}\Delta h$$

where ${\Delta \sigma }_{z}^{^{\prime}}$ is the variation of vertical effective stress, ${\gamma }_{w}$ is the water unit weight, and *∆h* is the changes in groundwater level. The compression of the soil layer is defined as^70^:

6 $${S}_{t}={m}_{v} \cdot {\Delta \sigma }_{z}^{^{\prime}} \cdot H=\frac{{S}_{sk}}{{\gamma }_{w}}\cdot{\Delta \sigma }_{z}^{^{\prime}} \cdot H={S}_{sk} \cdot \Delta h \cdot H={S}_{s} \cdot \Delta h$$

where *S*_*t*_ denotes the subsidence or the compression of soil layer (m), *m*_*v*_ is the coefficient of volume compressibility (Pa^−1^), *H* denotes the thickness of deformable soil (m), *S*_*sk*_ is the specific storage coefficient of the aquitard (m^−1^) called the skeletal specific storage, and *S*_*s*_ is the storage coefficient (dimensionless) of the aquitard. As seen in Eq. (6), *S*_*s*_ can be obtained by the calculation of inverse slope by plotting *∆h* versus *S*_*t*_.

Utilizing the InSAR subsidence rates and piezometric levels, the following simplified procedure is proposed to estimate the specific storage. It is important to note that this procedure can only be used in regions with large amounts of groundwater extraction and continuous withdrawals with a relatively linear trend. This approach can be applied particularly in fine-grained deformable thick soil layers due to soil consolidation which is a time-consuming phenomenon and results in a delay between the groundwater extraction and ground subsidence. The proposed method in this research estimates the specific storage with the following steps:

1. Selecting the piezometers in the study area with known piezometric groundwater levels. In this work, we have selected four well points, named W1, W2, W3, and W4.
2. Finding the trend in piezometric level time series by plotting the water level of the chosen wells versus time and calculating the water level change (*∆h*) in a time period (*∆t*). The slope of trend lines (dashed lines in Fig. 6) represents the average annual variation in the water level (*∆h/∆*t) for each well.
3. Finding the average rate of water fluctuation by plotting the piezometric level change (from Step 2) versus subsidence. As shown in Fig. 8, the inverse slope of the trend line shows the specific storage (*S*_*s*_) estimation.

**Figure 8:**
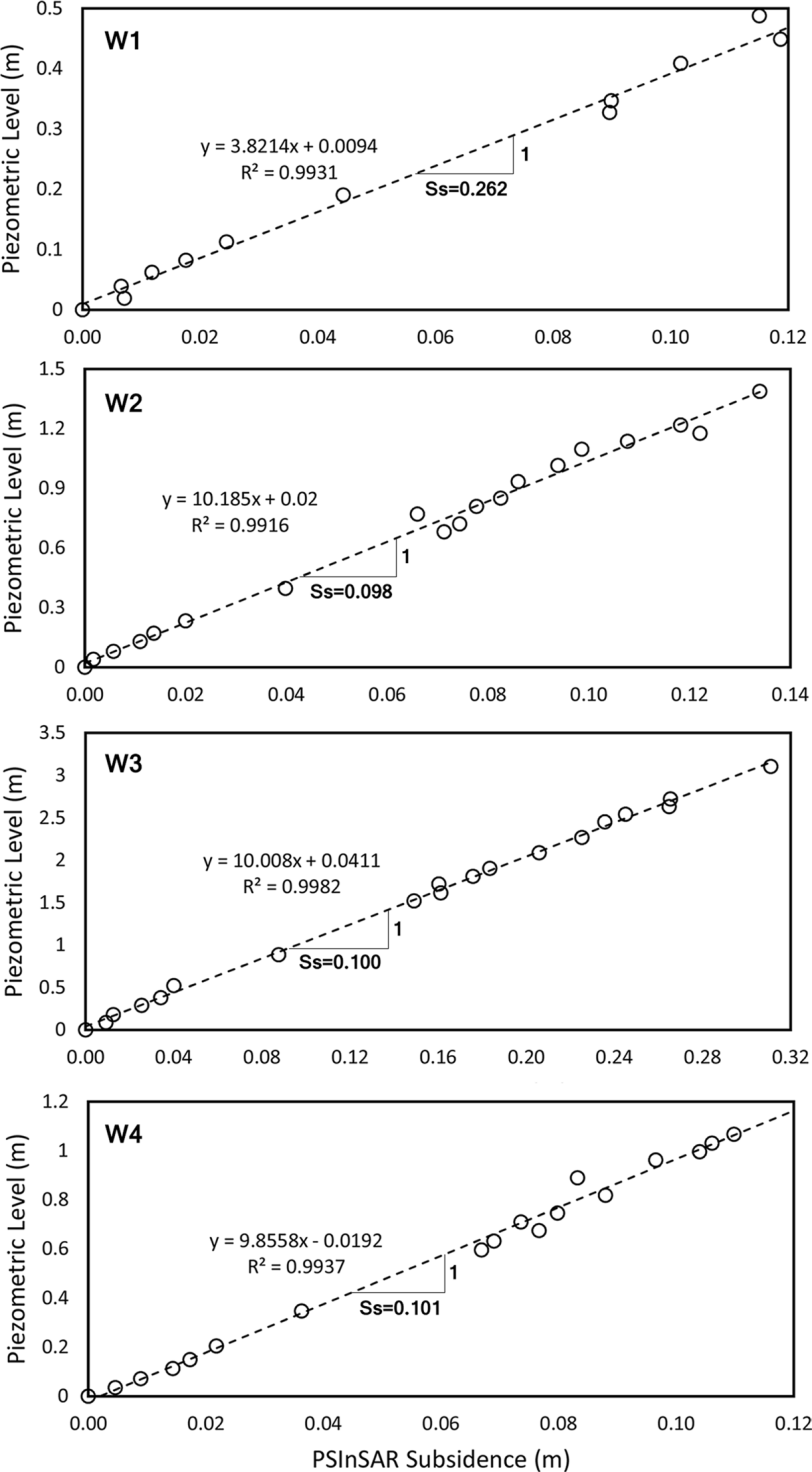
Piezometric level versus subsidence obtained by PSI to estimate specific storage (inverse slope of the trend line).
4. Computing the subsidence rate (${\dot{S}}_{t}$) by the estimated specific storage, using the following equation:
  7 $${\dot{S}}_{t}={S}_{s}\times \frac{\Delta h}{\Delta t}$$
  where $\Delta h$ is water level change and $\Delta t$ is time period. *∆h/∆t* and *S*_*s*_ are calculated in Step 2 and Step 3, respectively.

It should be noted that the second step above is the major part of the proposed approach which is superior to the previous method of specific storage estimation where water level variations are considered in the same period with the time of subsidence occurrence. However, the effect of water level variations on subsidence may have a significant delay depending on soil characteristics. Therefore, the period between 1990 to 2017 is considered for variations in groundwater level and a period between 2014 and 2017 for subsidence calculation. Then, we used average annual variation in the water level for each well. Finally, to compute specific storage, average piezometric level change has been plotted against the subsidence. Here, an important question arises: what is the suitable time period to consider water level variations? The answer to this question requires more comprehensive modeling and in-situ geotechnical data.

In Fig. 8, the trend lines (dashed lines) in the wells W1, W2, and W3 are in relative agreement with the real water level variations and demonstrate a continuous decline in groundwater level during the period. This trend was the reason to introduce this approach in the estimation of specific storage as there are no such obvious seasonal cycles. In the case of W4, it should be noted that the whole period shows a continuous decline in the groundwater level. Thus, it is selected in order to investigate the performance of our proposed framework. To estimate the specific storage in each well, we have drawn the output of Step 4 against the subsidence (which obtained by PSI). The inverse slope of this trend line (Fig. 8), which calculated in Table 5, shows the estimation for specific storages.

**Table 5:** Comparison between estimated subsidence rate by specific storage and PSI method.

Well	Estimated S_s_	∆h (m)	∆t (year)	∆h/∆t (m/year)	Subsidence by S_s_ (mm/year)	Subsidence by PSI (mm/year)
W1	0.262	0.59	1.94	0.30	78.6	78.5
W2	0.098	1.39	2.21	0.63	61.7	61.3
W3	0.100	3.10	2.21	1.41	141.0	140.0
W4	0.101	1.22	2.21	0.55	55.5	55.7

Table 5 shows the computed specific storage for each well separately and then based on the suggested procedure, the subsidence was calculated in the location of wells. Finally, the predicted subsidence compared to the PSI outputs in the same location. The benefit of PSI-based *S*_*s*_ is the high accuracy of its estimation. As the PSI method and piezometers provide the subsidence rate and water level decently accurate, the PSI-based *S*_*s*_ can be considered more reliable than other existing methods. So, the proposed method can be used in similar cases in future studies to compute the specific storage and accordingly to predict ground subsidence.

## Conclusions

Mashhad, as one of the largest and most populated cities in the Middle East, has been suffering from extreme subsidence mostly due to groundwater over-extraction. In the past decade, several researchers have shown interest in measuring land subsidence rates in the Mashhad valley by InSAR techniques. Lack of high-resolution images and, consequently, imperfect measurements, introduces uncertainties in the reported subsidence rates in this area. Most, if not all, of the published works for this region, used a limited number of EnviSat data with long perpendicular and inhomogeneous temporal baseline. This paper determined the subsidence rate in Mashhad in recent years and for this purpose, the PSI technique was applied in the study area using two time-series of descending and ascending Sentinel-1A acquisitions between 2014 and 2017. The results demonstrated the maximum line-of-sight deformation rate of 14.6 cm/year and maximum vertical deformation (subsidence) rate of 19.1 cm/year. The results were assessed and validated using piezometric data, GPS stations, and geotechnical properties. Lastly, a new simplified method was proposed to estimate specific storage for subsidence rate prediction. The main conclusions of the present study can be drawn as follow:

- The results of this study show that ground subsidence is continuous particularly in the northwest of the analysis area, suffering from the maximum vertical subsidence rate of 19.1 cm/year for the 2014–2017 period. The results include the extent of subsidence area around the maximum deformation rate, which covers a large part of the northwest of the city, which may have damages and irreversible consequences in the future.
- The PSI study of subsidence in Mashhad shows that groundwater drawdown rate and geotechnical properties in the area, both strongly influence the rate and the distribution of subsidence. This issue (high subsidence rate) can not be investigated solely on the basis of water level changes, and any interpretation in this regard requires more geotechnical information. But, in areas of high alluvial thickness such as Mashhad, geotechnical data extraction from bedding is time-consuming and costly. The specific storage parameter enables an initial estimation/prediction of subsidence without the need for accurate geotechnical information.
- A new simplified method is proposed to estimate specific storage for areas with significant alluvial thickness and a high rate of groundwater extraction. This approach is developed in order to overcome the great delay between the groundwater extraction and its corresponding ground subsidence. A proper correlation between the subsidence values and the average water level indicates that the proposed method is reliable. Also, the utilization of PSI provides an accurate estimation of specific storage and subsidence rate for a large study area. The proposed method can be used in similar cases in future studies to compute the specific storage and accordingly to predict ground subsidence.
- Collecting deep geotechnical boreholes indicates thick fine-grained layers in the northwest of the city which have not been reported by past researchers. This finding confirms the high subsidence rates in the northwest of Mashhad due to a large amount of groundwater extraction and corresponding consolidation phenomenon in fine-grained layers. This observation indicates the necessity of more detailed geotechnical investigations in the area of interest.

## Data Availability

All of the analyzed groundwater level data, geotechnical data, and GPS observations collected during this study are included in this article. Sentinel-1 data are accessible through the Copernicus Open Data Hub. The generated datasets of this study are available from the corresponding author on reasonable request.
